# A brief-review of the risk factors for covid-19 severity

**DOI:** 10.11606/s1518-8787.2020054002481

**Published:** 2020-05-28

**Authors:** J.E. Rod, Oscar Oviedo-Trespalacios, Javier Cortes-Ramirez

**Affiliations:** I Queensland University of Technology School of Public Health and Social Work BrisbaneQueensland Australia Queensland University of Technology (QUT). School of Public Health and Social Work. Brisbane, Queensland, Australia; II Queensland University of Technology Institute of Health and Biomedical Innovation BrisbaneQueensland Australia Queensland University of Technology (QUT). Institute of Health and Biomedical Innovation. Brisbane, Queensland, Australia; III Queensland University of Technology Centre for Accident Research and Road Safety Queensland BrisbaneQueensland Australia Queensland University of Technology (QUT). Centre for Accident Research and Road Safety Queensland (CARRS-Q). Brisbane, Queensland, Australia; IV Universidad del Norte Departamento de ingeniería BarranquilaAtlántico Colombia Universidad del Norte. Departamento de ingeniería. Barranquila, Atlántico, Colombia

**Keywords:** Coronavirus Infections, epidemiology, Fatal Outcome, Risk Factors, Review

## Abstract

The World Health Organization has emphasized that one of the most important questions to address regarding the covid-19 pandemic is to understand risk factors for disease severity. We conducted a brief review that synthesizes the available evidence and provides a judgment on the consistency of the association between risk factors and a composite end-point of severe-fatal covid-19. Additionally, we also conducted a comparability analysis of risk factors across 17 studies. We found evidence supporting a total of 60 predictors for disease severity, of which seven were deemed of high consistency, 40 of medium and 13 of low. Among the factors with high consistency of association, we found age, C-reactive protein, D-dimer, albumin, body temperature, SOFA score and diabetes. The results suggest that diabetes might be the most consistent comorbidity predicting disease severity and that future research should carefully consider the comparability of reporting cases, factors, and outcomes along the different stages of the natural history of covid-19.

## INTRODUCTION

More than 200 countries and territories have reported confirmed cases of the novel coronavirus disease covid-19, characterized as a pandemic by the World Health Organisation on April 7, 2020^1^. As this global health emergency tests the resilience of health systems around the world, health care and public health practitioners are required to have high quality evidence to identify its most significant risks and prioritize resources where they are most needed.

One of the most important questions to address the currently unfolding pandemic is *“*what are the risk factors for severe illness or death?”^1,2^. Systematic reviews and meta-analysis paired with a standardised method to assess the quality of evidence are deemed to provide the best evidence by current standards^3^. For example, the GRADE evidence assessment focuses on comparing factors or outcomes across studies to provide an evidence synthesis. If researchers conduct a GRADE evaluation for covid-19 severity in the future, the time investment and internal validity of the assessment will be heavily influenced by the consistency of categorizations and reporting of cases, factors, and outcomes across studies.

Since scientific publications addressing the pandemic are being produced rapidly, including risk factor studies, summarizing and sharing such information is of paramount importance to support an efficient and rapid response. An early review of risk factor studies could also provide some insight on the undesirable heterogeneity of definitions and reporting that might affect later evidence evaluations. Therefore, we compiled a brief summary of the literature evaluating the risk factors for covid-19 disease severity with a two-fold purpose: (i) to provide healthcare and public health professionals with a reference list of the consistency of risk factors for covid-19 severity, and (ii) to inform researchers about the consistency of reporting in the available literature.

## METHODS

We conducted a review to assess studies looking for risk factors of severity or death for covid-19, using a composite outcome of disease severity-fatality (CSF)^4^. Unstructured searches using the terms: “disease attributes,” “clinical findings,” “clinical features,” “clinical characteristic,” “novel coronavirus,” “covid-19,” “SARS-Cov-2,” “fatality,” “fatal,” “death,” “mortality,” “severity,” “disease severity,” “predictor,” and “risk factor” were performed to identify articles written in English available on PubMed, Scopus and MedRxiv. Articles were selected for the review if they included a comparison between non-severe and CSF cases according to the categorization of severity in each article. After completing article selection, we assessed the consistence of statistically significant associations for a particular risk factor by classifying it as high, medium, and low, following two criteria: (i) a positive difference in the total number of studies reporting statistical significance dissimilarities between non-severe and CSF, minus the number of studies without statistical significance for the same factor, and, (ii) the reporting of statistically significant estimates when performing multivariate statistics. The consistency of association was categorized as high (both criteria were met), medium (one criterion), low (none). Additionally, we assessed heterogeneity by comparing the terminology, units, statistical descriptions, and cut-off points of each risk factor and reported the highest comparable (hc) number across studies. We then subtracted the total of studies including the risk factor by the hc number across the sample (Table).

**Table:** Summary of risk factors associated with covid-19 severity and evaluation of reporting consistency

RISK FACTORS	Consistency computation for each factor			Studies not reporting *p* values *(n)*	Total (*p* < 0.05) - total (*p* > 0.05)	Results of (*p* < 0.05) multivariate statistics	Consistency	Heterogeneity
	
	All studies *(n)* with *p* < 0.05 for differences non-severe vs CSF	% of *p* < 0.05 studies supporting a value direction or the presence of the risk factor	Studies *p* > 0.05 (n)				●●● High ●●○ Medium ●○○ Low	hc# (Total - hc#/total) hc-statistic hc-definition, unit
Age	Zhang et al.^6^ (19) Wang et al.^7^ (138) Tian et al.^8^ (262) Liu et al.^9^ (109) Liu et al.^10^ (78) Li et al.^11^ (84) Caramelo et al.^12^ (72314) Tang et al.^13^ (183) Ruan et al.^14^ (150) Zhou et al.^15^ (191) Wu et al.^16^ (201)	100% - ↑	Huang et al.^17^ (41)	Guan et al.^18^ (1099) Yang et al.^19^ (52) Young et al.^20^ (18) Ji et al.^21^ (NA) Zhonghua^22^ (44672)	10	Liu et al.^10^ ≥ 60: OR 8.5 95% CI 1.6 - 44.8 Caramelo et al.^12^ 50-59: OR 6.7 95% CI 2.9 - 15.2 ≥ 80: OR 86.6 95% CI 32.6 - 202.4 Zhou et al.^15^ NA: OR 1.1 95% CI 1.0 - 1.2	●●●	12 (5/17) = 29% Median (IQR) Age, years
C- reactive protein	Zhang et al.^6^ (19) Li et al.^11^ (84) Liu et al.^10^ (78) Liu et al.^9^ (109) Ruan et al.^14^ (150) Wu et al.^16^ (201)	100% - ↑	0	Young et al.^20^ (18) Guan et al.^18^ (1099)	6	Liu et al.^10^ > 8.2 mg/L: OR 10.5 95% CI 1.2 - 34.7	●●●	6 (2/8) = 25% Median (IQR) C-reactive protein, mg/L
D- Dimer	Tang et al.^13^ (183) Huang et al.^17^ (41) Zhang et al.^6^ (19) Wang et al.^7^ (138) Liu et al.^9^ (109) Zhou et al.15 (191) Wu et al.^16^ (201)	100% - ↑	Liu et al.^10^ (78)	Guan et al.^18^ (1099)	6	Zhou et al.^15^ > 1 μg/mL: OR 18.42 95% CI 2.6 - 128.5	●●●	7 (2/9) = 22% Median (IQR) D-dimer, μg/mL
Albumin	Liu et al.^10^ (78) Huang et al.^17^ (41) Ruan et al.^14^ (150) Zhou et al.15 (191) Wu et al.^16^ (201)	100% - ↓	0	0	5	Liu et al.^10^ < 40 g/L: OR 7.4 95% CI 1.1 - 50.0	●●●	4 (1/5) = 20% Median (IQR) Albumin, g/L
Body temperature	Huang et al.^17^ (41) Li et al.^11^ (84) Liu et al.^10^ (78) Wu et al.^16^ (201)	75% - ↑	Tian et al.^8^ (262)	Guan et al.^18^ (1099) Young et al.^20^ (18)	3	Liu et al.^10^ ≥ 37.3°: OR 8.9 95% CI 1.0 - 78.1	●●●	4 (3/7) = 43% Median (IQR) Highest temperature, C°
SOFA score	Liu et al.^9^ (109) Zhou et al.^15^ (191)	100% - ↑	0	0	2	Zhou et al.^15^ NA: OR 5.7 95% CI 2.6 - 12.2	●●●	2 (0/2) = 0% Median (IQR) SOFA score, NA
Diabetes	Caramelo et al.^12^ (72314) Li et al.^11^ (84) Wang et al.^7^ (138) Liu et al.^9^ (109) Zhou et al^.15^ (191) Wu et al.^16^ (201)	100% - Presence	Huang et al.^17^ (41) Zhang et al.^6^ (19) Liu et al.^10^ (78) Ruan et al.^14^ (150)	Guan et al.^18^ (1099) Zhonghua^22^ (44672) Yang et al.^19^ (52)	2	Caramelo et al.^12^ Diabetes: OR 9.0 95% CI 7.4 - 11.3	●●●	13 (0/13) = 0% Percentages Diabetes, NA
Lymphocyte count	Zhang et al.^6^ (19) Li et al.^11^ (84) Wang et al.^7^ (138) Huang et al.^17^ (41) Liu et al.^9^ (109) Ruan et al.^14^ (150) Wu et al.^16^ (201) Zhou et al.^15^ (191)	100% - ↓	Yang et al.^19^ (52) Liu et al.^10^ (78)	Young et al.^20^ (18) Guan et al.^18^ (1099)	6	NA	●●○	9 (3/12) = 25% Median (IQR) Lymphocyte count, ×109/L
Dyspnea	Li et al.^11^ (84) Huang et al.^17^ (41) Tian et al.^8^ (262) Wang et al.^7^ (138) Ruan et al.^14^ (150) Wu et al.^16^ (201)	100% - Presence	Zhang et al.^6^ (19)	Yang et al.^19^ (52) Guan et al.^18^ (1099) Young et al.^20^ (18)	5	NA	●●○	10 (0/10) = 0% Percentages Dyspnea, NA
White blood Cell count	Huang et al.^17^ (41) Wang et al.^7^ (138) Liu et al.^9^ (109) Ruan et al.^14^ (150) Zhou et al.^15^ (191) Wu et al.^16^ (201) Zhang et al.^6^ (19)	100% - ↑	Li et al.^11^ (84) Liu et al.^10^ (78)	Guan et al.^18^ (1099) Young et al.^20^ (18)	5	NA	●●○	8 (3/11) = 27% Median (IQR) WBC count, ×109/L
Procalcitonin	Li et al.^11^ (84) Wang et al.^7^ (138) Huang et al.^17^ (41) Liu et al.^9^ (109) Zhou et al.^15^ (191)	100% - ↑	Liu et al.^10^ (78)	Guan et al.^18^ (1099)	4	NA	●●○	6 (1/7) = 14% Median (IQR) Procalcitonin, ng/ml
Lactate dehydrogenase	Wang et al.^7^ (138) Huang et al.^17^ (41) Liu et al.^9^ (109) Zhou et al.^15^ (191) Wu et al.^16^ (201)	100% - ↑	Ruan et al.^14^ (150)	Young et al.^20^ (18) Guan et al.^18^ (1099)	4	NA	●●○	7 (1/8) = 13% Median (IQR) LDH, U/L
Cardiac troponins	Wang et al.^7^ (138) Huang et al.^17^ (41) Ruan et al.^14^ (150) Zhou et al.^15^ (191)	100% - ↑	0	0	4	NA	●●○	3 (1/4) = 25% Median (IQR) Hypersensitive troponin I, pg/ml
Prothrombin time	Huang et al.^17^ (41) Tang et al.^13^ (183) Zhou et al.^15^ (191) Wu et al.^16^ (201)	100% - ↑	Wang et al.^7^ (138)	Yang et al.^19^ (52)	3	NA	●●○	5 (1/6) = 17% Median (IQR) Prothrombin time, s,
Blood urea nitrogen	Ruan et al.^14^ (150) Liu et al.^9^ (109) Wang et al.^7^ (138)	100% - ↑	0	0	3	NA	●●○	3 (0/3) = 0% Blood urea nitrogen, mmol/L
Total bilirubin	Ruan et al.^14^ (150) Wang et al.^7^ (138) Huang et al.^17^ (41) Wu et al.^16^ (201)	100% - ↑	Liu et al.^9^ (109)	Yang et al.^19^ (52) Guan et al.^18^ (1099)	3	NA	●●○	4 (3/7) = 43% Total bilirubin, μmol/L
Interleukin-6	Ruan et al.^14^ (150) Zhou et al.^15^ (191) Wu et al.^16^ (201)	100% - ↑	0	0	3	NA	●●○	3 (0/3) = 0% Median (IQR) IL-6, pg/mL
Serum ferritin	Ruan et al.^14^ (150) Zhou et al.^15^ (191) Wu et al.^16^ (201)	100% - ↑	0	0	3	NA	●●○	3 (0/3) = 0% Median (IQR) Serum ferritin, ng/mL
Comorbidity	Li et al.^11^ (84) Wang et al.^7^ (138) Zhang et al.^6^ (19) Ruan et al.^14^ (150) Zhou et al.^15^ (191)	100% - Presence	Huang et al.^17^ (41) Tang et al.^13^ (183)	Guan et al.^18^ (1099) Yang et al.^19^ (52)	3	NA	●●○	8 (0/8) = 0% Percentages Comorbidity, NA
Neutrophil count	Huang et al.^17^ (41) Wang et al.^7^ (138) Liu et al.^9^ (109) Wu et al.^16^ (201)	75% - ↑	Liu et al.^10^ (78) Li et al.^11^ (84)	Young et al.^20^ (18)	2	NA	●●○	7 (0/7) = 0% Median (IQR) Neutrophil count, ×109/L
Creatine kinase MB	Wang et al.^7^ (138) Zhou et al.^15^ (191) Wu et al.^16^ (201)	100% - ↑	Liu et al.^9^ (109)	0	2	NA	●●○	4 (0/4) = 0% Median (IQR) CK-MB, U/L
CURB-65	Liu et al.^9^ (109) Zhou et al.^15^ (191)	100% - ↑	0	0	2	NA	●●○	2 (0/2) = 0% Median (IQR) CURB-65 score, NA
Respiratory rate	Huang et al.^17^ (41) Liu et al.^10^ (78) Tian et al.^8^ (262) Zhou et al.^15^ (191)	100% - ↑	Li et al.^11^ (84) Wang et al.^7^ (138)	Young et al.^20^ (18)	2	NA	●●○	3 (4/7) = 57% Median (IQR) Respiratory rate, breaths*min
Lymphocyte ratio	Li et al.^11^ (84)	100% - ↓	0	0	1	NA	●●○	1 (0/1) = 0% Mean (SD) Lymphocyte ratio, %
Chronic kidney disease	Liu et al.^9^ (109) Zhou et al.^15^ (191)	100% - Presence	Ruan et al.^14^ (150)	0	1	NA	●●○	3 (0/3) = 0% Percentages Chronic kidney dis ease, NA
Chest pain	Li et al.^11^ (84)	100% - Presence	0	Yang et al.^19^ (52)	1	NA	●●○	2 (0/2) = 0% Percentages Chest pain, NA
Neutrophil ratio	Li et al.^11^ (84)	100% - ↑	0	0	1	NA	●●○	1 (0/1) = 0% Percentage Neutrophil ratio, %
Fibrinogen degradation product	Tang et al.^13^ (183)	100% - ↑	0	0	1	NA	●●○	1 (0/1) = 0% Median (IQR) FDP, ug/mL
Myoglobin	Ruan et al.^14^ (150)	100% - ↑	0	0	1	NA	●●○	1 (0/1) = 0% Mean (SD) Myoglobin, ng/mL
APACHE II	Liu et al.^9^ (109)	100% - ↑	0	0	1	NA	●●○	1 (0/1) = 0% Median (IQR) APACHE II score, NA
PaO2:FiO2	Liu et al.^9^ (109)	100% - ↓	0	0	1	NA	●●○	1 (0/1) = 0% Median (IQR) PaO2:FiO2, mmHg
Globulin	Wu et al.^16^ (201)	100% - ↑	0	0	1	NA	●●○	1 (0/1) = 0% Median (IQR) Globulin, g/L
Prealbumin	Wu et al.^16^ (201)	100% - ↓	0	0	1	NA	●●○	1 (0/1) = 0% Median (IQR) prealbumin, mg/L
Urea	Wu et al.^16^ (201)	100% - ↑	0	0	1	NA	●●○	1 (0/1) = 0% Median (IQR) prealbumin, mM
Glucose	Wu et al.^16^ (201)	100% - ↑	0	0	1	NA	●●○	1 (0/1) = 0% Median (IQR) Glucose, mM
Cholinesterase	Wu et al.^16^ (201)	100% - ↓	0	0	1	NA	●●○	1 (0/1) = 0% Median (IQR) Cholinesterase, U/L
Cystatin C	Wu et al.^16^ (201)	100% - ↑	0	0	1	NA	●●○	1 (0/1) = 0% Median (IQR) Cystatin C, mg/L
α-HBDH	Wu et al.^16^ (201)	100% - ↑	0	0	1	NA	●●○	1 (0/1) = 0% Median (IQR) α-HBDH 100 U/L
LDL	Wu et al.^16^ (201)	100% - ↓	0	0	1	NA	●●○	1 (0/1) = 0% Median (IQR) LDL, mM
Heart Rate	Zhou et al.^15^ (191)	100% - ↑	0	0	1	NA	●●○	1 (0/1) = 0% Percentages Heart Rate ≥125, beats/min
Health system burden in Hubei	Ji et al.^21^ (NA)	100% - ↑	0	0	1	NA	●●○	1 (0/1) = 0% NA NA
Days from onset of symptoms to hospital	Li et al.^11^ (84) Wang et al.^7^ (138) Tian et al.^8^ (262)*	100% - ↑	Zhang et al.^6^ (19) Zhou et al.^15^ (191)	Huang et al.^17^ (41)	1	NA	●●○	5 (1/6) = 17% Median (IQR) Symptom onset to admission, days
O2 saturation	Li et al.^11^ (84)	100% - ↓	Liu et al.^10^ (78)	Young et al.^20^ (18)	0	NA	●●○	3 (0/3) = 0% Median (IQR) Oxygen saturation, %
Fibrinogen	Liu et al.^9^ (109)	100% - ↑	Tang et al.^13^ (183)	0	0	NA	●●○	2 (0/2) = 0% Median (IQR) Fibrinogen, g/L
Smoking	Liu et al.^10^ (78)	100% - Presence	Huang et al.^17^ (41) Zhang et al.^6^ (19) Zhou et al.^15^ (191)	Guan et al.^18^ (1099) Yang et al.^19^ (52)	-2	Liu et al.^10^ Past use: OR 14.3 95% CI 1.6 - 25.0	●●○	5 (1/6 = 17%) Percentages Current smoker, NA
Chronic respiratory disease	Caramelo et al.^12^ (72314) Li et al.^11^ (84) Zhou et al.^15^ (191)	100% - Presence	Huang et al.^17^ (41) Zhang et al.^6^ (19) Wang et al.^7^ (138) Liu et al.^10^ (78) Liu et al.^9^ (109) Ruan et al.^14^ (150)	Yang et al.^19^ (52) Zhonghua^22^ (44672) Guan et al.^18^ (1099)	-3	Caramelo et al.^12^ CRD: OR 7.8 95% CI 5.5 - 10.4	●●○	10 (2/12 = 17%) Percentages COPD, NA
Cancer	Caramelo et al.^12^ (72314)	100% - Presence	Huang et al.^17^ (41) Liu et al.^10^ (78) Ruan et al.^14^ (150) Zhou et al.^15^ (191)	Zhonghua^22^ (44672) Guan et al.^18^ (1099)	-3	Liu et al.^10^ Cancer: OR 6.9 95% CI 3.4 - 12.5	●●○	7 (0/7) = 0% Percentages Malignancy, NA
Cardiovascular disease	Caramelo et al.^12^ (72314) Ruan et al.^14^ (150) Zhou et al.^15^ (191)	100% - Presence	Huang et al.^17^ (41) Wang et al.^7^ (138) Liu et al.^9^ (109) Zhang et al.^6^ (19) Liu et al.^10^ (78) Li et al.^11^ (84) Wu et al.^16^ (201)	Guan et al.^18^ (1099) Yang et al.^19^ (52) Zhonghua^22^ (44672)	-4	Caramelo et al.^12^ HTA: OR 7.4 95% CI 6.3 - 8.8 Cardiac: OR 12.8 95% CI 10.3 - 15.9	●●○	12 (1/13) =8% Percentages Cardiac disease, NA
Lactate	Liu et al.^9^ (109)	100% - ↑	0	Yang et al.^19^ (52) Wang et al.^7^ (138)	-1	NA	●○○	3 (0/3) = 0% Median (IQR) Lactate, mmol/L
Monocyte count	Li et al.^11^ (84)	100% - ↓	Wang et al.^7^ (138) Wu et al.^16^ (201)	0	-1	NA	●○○	3 (0/3) = 0% Median (IQR) Lymphocite count / ×109/L
Creatinine	Ruan et al.^14^ (150) Wang et al.^7^ (138) Zhou et al.^15^ (191)	100% - ↑	Liu et al.^10^ (78) Huang et al.^17^ (41) Liu et al.^9^ (109) Wu et al.^16^ (201)	Yang et al.^19^ (52) Guan et al.^18^ (1099)	-1	NA	●○○	5 (4/9) = 44% Median (IQR) Creatinin, μmol
Composite abnormal radiological findings (CT-RX)	Li et al.^11^ (84) Zhou et al.^15^ (191)	100% - ↑	Wang et al.^7^ (138) Zhang et al.^6^ (19) Huang et al.^17^ (41)	Young et al.^20^ (18) Guan et al.^18^ (1099)	-1	NA	●○○	0 (6/6) = 100% NA NA
Systolic blood pressure	Huang et al.^17^ (41)	100% - ↑	Tian et al.^8^ (262)* Zhou et al.^15^ (191)	Yang et al.^19^ (52) Young et al.^20^ (18)	-1	NA	●○○	2 (2/5) = 40% Mean (SD) Median (IQR) Systolic blood pressure, mmHg
Platelet count	Ruan et al.^14^ (150) Zhou et al.^15^ (191)	100% - ↓	Wang et al.^7^ (138) Liu et al.^10^ (78) Wu et al.^16^ (201)	Yang et al.^19^ (52) Tang et al.^13^ (183) Young et al.^20^ (18) Guan et al.^18^ (1099) Huang et al.^17^ (41)	-1	NA	●○○	5 (5/10) = 50% Median (IQR) Platelet count / ×109/L
AST	Wang et al.^7^ (138) Wu et al.^16^ (201)	100% - ↑	Liu et al.^10^ (78) Liu et al.^9^ (109) Ruan et al.^14^ (150)	Guan et al.^18^ (1099) Huang et al.^17^ (41)	-1	NA	●○○	6 (1/7) = 14% Median (IQR) Aspartate aminotransferase, UL
ALT	Wang et al.^7^ (138) Huang et al.^17^ (41) Zhou et al.^15^ (191)	100% - ↑	Liu et al.^10^ (78) Liu et al.^9^ (109) Ruan et al.^14^ (150) Wu et al.^16^ (201)	Guan et al.^18^ (1099)	-1	NA	●○○	6 (2/8) = 25% Median (IQR) Alanine aminotransferase, UL
Expectoration	Li et al.^11^ (84)	100% - Presence	Wang et al.^7^ (138) Huang et al.^17^ (41) Ruan et al.^14^ (150) Zhou et al.^15^ (191)	Guan et al.^18^ (1099)	-3	NA	●○○	6 (0/6) = 0% Percentages Sputum, NA
Cough	Li et al.^11^ (84) Zhang et al.^6^ (19)	100% - Presence	Huang et al.^17^ (41) Tian et al.^8^ (262) Wang et al.^7^ (138) Liu et al.^10^ (78) Liu et al.^9^ (109) Ruan et al.^14^ (150) Zhou et al.^15^ (191) Wu et al.^16^ (201)	Yang et al.^19^ (52) Guan et al.^18^ (1099) Young et al.^20^ (18)	-6	NA	●○○	11 (2/13) = 15% Percentages Cough, NA
Fatigue	Liu et al.^9^ (109)	100% - Presence	Huang et al.^17^ (41) Zhang et al.^6^ (19) Tian et al.^8^ (262) Wang et al.^7^ (138) Ruan et al.^14^ (150) Wu et al.^16^ (201) Zhou et al.^15^ (191)	Guan et al.^18^ (1099)	-6	NA	●○○	5 (2/7) = 29% Percentages Fatigue, NA
Male gender	Tang et al.^13^ (183) Caramelo et al.^12^ (72314)	100% - Presence	Huang et al.^17^ (41) Zhang et al.^6^ (19) Wang et al.^7^ (138) Tian et al.^8^ (262) Liu et al.^9^ (109) Li et al.^11^ (84) Ruan et al.^14^ (150) Liu et al.^10^ (78) Zhou et al.^15^ (191) Wu et al.^16^ (201)	Guan et al.^18^ (1099) Yang et al.^19^ (52) Zhonghua^22^ (44672) Young et al.^20^ (18)	-8	NA	●○○	15 (1/16) = 6% Percentages Male, NA

HTA: hypertension; CRD: chronic respiratory disease

* Data provided directly by the authors of the publication.

## RESULTS

We identified a total of 17 studies, with most of them relying on a retrospective cross-sectional design and reporting data using descriptive statistics. Only three studies performed multivariate analysis adjusting for confounding factors. Sixteen of the studies reported laboratory-confirmed cases of covid-19 and one reported clinically diagnosed cases. There were 60 risk factors identified for COVID-19 severity (Table). Of these, 7 were considered of high, 40 of medium and 13 of low consistency. Increasing values of age, D-dimer, C-reactive protein, sequential organ failure assessment (SOFA) score and body temperature while decreasing albumin, and a history of diabetes were the risk factors with the highest consistency as predictors for covid-19 severity. Additionally, elevated values of white blood cells count, procalcitonin, lactate dehydrogenase, cardiac troponins, prothrombin time, interleukin-6, serum ferritin, neutrophils count, creatine kinase MB, CURB-65 score with decreased lymphocyte count, and dyspnea were classified as medium consistency risk factors with at least a positive difference of two studies reporting a statistically significant difference between non-severe and CSF groups. There was high heterogeneity in the definition of CSF, ranging from the need for supplemental oxygen to the development of acute respiratory distress syndrome (ARDS), ICU admission and death. In terms of risk factor heterogeneity, 40% of factors presented a value of zero with an overall median of 14% (IQR = 0–25). Nevertheless, when considering only the remaining 60% variables, the mean heterogeneity value was 28.5% (SD = 19.6).

## DISCUSSION

The results from this review are consistent with current analyses considering age and comorbidities the most important risk factors for covid-19 disease severity. However, our findings also suggest that diabetes is one of the most critical comorbidities in terms of disease severity. Diabetes has been previously associated with other respiratory virus disease severities in cross national samples^5^. This might be explained by the immunosuppressive effects of hyperglycaemia^5^ and could also explain why patients that develop ARDS due to covid-19 were found to have statistically significant higher glucose levels (Table). This finding has important implications given the high global prevalence of diabetes. When considering the heterogeneity of reported factors across studies, 60% presented some level of heterogeneity, which indicates that there is a need for higher reporting consistency in future research looking at the risk factors for covid-19 disease severity.

Some limitations should be considered when interpreting these findings. Most of the selected studies were conducted in China (and one in Singapore), limiting the external validity of risk factors for other countries. As we used a composite index of severity, the relevance of these factors varies according to the natural history of the disease with some factors, such as body temperature and neutrophils count (and their value directions -higher/lower) being more relevant for different stages. A limitation of this study is the rapid growth of knowledge about covid-19. Therefore, the results of the present review might vary as the scientific understanding of covid-19 progresses. Additionally, two of the 17 studies reviewed were pre-prints (neither published, nor peer-reviewed). Despite this, the wide range of risk factors identified across 17 publications can guide future research to rapidly validate the present results on cross-national samples.

Given that the burden to the health system due to covid-19 is a determinant of the disease severity (Table), the results from this review can support clinical and public health initiatives to target populations and patients that are at most risk while further evidence is generated. Additionally, the present review provides researchers with a rapid reference on reported clinical and demographical factors in order to increase the comparability of results and further decrease uncertainties regarding the covid-19 severity. This can support clinical management decisions and the design of strategies to inform the general public about important risk factors for covid-19 severity, For instance, when communicating who is most at risk for the disease, instead of making a broad generalization such as “increasing age and underlying health conditions,” messaging that communicates risk factors should at a minimum include: age > 50, diabetes, smoking, respiratory disease, cancer and cardiovascular disease. Additionally, for patients that are isolated outside a healthcare institution either due to clinical suspicion or confirmed mild case, specific factors such as shortness of breath and chest pain could be communicated as triggers for seeking care. Factors such as fatigue, cough and expectoration have low consistency for predicting disease severity and by themselves should not be relied upon for clinical assessment. We expect that the results of this brief review can support government and medical strategies in response to the pandemic.
